# *Katatopygia* gen. n., a monophyletic branch segregated from Boletina (Diptera, Mycetophilidae)

**DOI:** 10.3897/zookeys.175.2388

**Published:** 2012-03-16

**Authors:** Svante Martinsson, Jostein Kjærandsen

**Affiliations:** 1 Department of Zoology, University of Gothenburg, Box 463, SE-405 30 Göteborg, Sweden; 2 Museum of Zoology, Department of Biology, Lund University, Helgonavägen 3, SE-223 62 Lund, Sweden

**Keywords:** New genus, taxonomy, Gnoristinae, new synonymy, revision, identification key, phylogeny

## Abstract

The genus *Katatopygia* **gen. n.** is proposed for the *Boletina erythropyga/punctus*-group that was first introduced by Garrett (1924, 1925) and currently comprises eight described species. Molecular studies have strongly indicated that this group forms a monophyletic sister-group to a clade consisting of all other *Boletina*, *Coelosia* and *Gnoriste*, and its monophyly is supported by morphological data as well. The new genus includes the following species: *Katatopygia antoma* (Garrett, 1924), **comb. n.**, *Katatopygia antica* (Garrett, 1924), **comb. n.**, *Katatopygia erythropyga* (Holmgren, 1883), **comb. n.**,*Katatopygia hissarica* (Zaitzev & Polevoi, 2002), **comb. n.**, *Katatopygia magna* (Garrett, 1925), **comb. n.**, *Katatopygia laticauda* (Saigusa, 1968), **comb. n.**, *Katatopygia neoerythropyga* (Zaitzev & Polevoi, 2002), **comb. n.** and*Katatopygia sahlbergi* (Lundström, 1906), **comb. n.**, all transferred from *Boletina*. *Katatopygia sahlbergi* is found to be a senior synonym of *Boletina punctus* Garrett, 1925, **syn. n.** A phylogeny based on morphological data and using parsimony analysis yielded four most parsimonious trees where the new genus is retrieved as monophyletic with high support. *Katatopygia neoerythropyga* is found to be the sister-taxon to all other species that form two clades, one with *Katatopygia sahlbergi*-like species and one with *Katatopygia erythropyga*-like species. A key to males of *Katatopygia* is provided.

## Introduction

In two papers Garrett (1924, 1925) described four closely allied species of *Boletina* Staeger from North America that he in the latter paper named the *punctus*-group and provided a sketchy plate with all four species’ male gonostyles aligned. Zaitzev and Polevoi (2002) revised the same group under the name *erythropyga*-group, but were apparently not aware of and failed to include Garrett’s species although their revision was intended to be Holarctic. Although traditionally placed in *Boletina*,species of this group are morphologically quite distinct in several aspects and well separated from other *Boletina* as well as from *Saigusaia* Vockeroth and *Aglaomyia* Vockeroth, both of which Vockeroth (1980) distinguished from *Boletina* based on morphological characters. The segregation of *Saigusaia* has later been supported by phylogenetic studies, both morphological (Søli 1997) and molecular (Martinsson et al. 2011), whereas *Aglaomyia* may be nested within *Boletina* (Martinsson et al. 2011). Molecular studies have strongly suggested that *Boletina* as currently delimited is paraphyletic (Baxter 1999; Martinsson et al. 2011) and given support for the *Boletina erythropyga/punctus*-group being the sister-group to a clade consisting of all other *Boletina*, *Aglaomyia*, *Coelosia* Winnertz and *Gnoriste* Meigen (Martinsson et al. 2011).

Accordingly, a new genus *Katatopygia* gen. n. is here proposed and described for the *Boletina erythropyga/punctus*-group. Holmgren (1883) described the first species as *Boletina erythropyga* Holmgren, 1883, based on material from Novaya Zemlya in northern Russia. Lundström (1906) added *Boletina sahlbergi* Lundström, 1906 from Finnish Lapland, while Johannsen (1911) added *Boletina longicornis* Johannsen, 1911 from Idaho in USA. Garrett (1924, 1925) described four additional species based on material from British Columbia in Canada, of which *Boletina punctus* syn. n. is considered here a junior synonym of *Boletina sahlbergi*. Saigusa (1968) added *Boletina laticauda* Saigusa, 1968 from Taiwan. Laštovka and Matile (1988) erroneously synonymized *Boletina sahlbergi* with *Boletina erythropyga*, leading Zaitzev (1994) to describe a new species representing the former. Zaitzev and Polevoi (2002) resolved these confusing species interpretations by reinstating *Boletina sahlbergi* and suggesting two new synonyms, and finally added two more species. With these changes a group that currently includes eight species can be assigned to *Katatopygia* gen. n. (Table 1), all being transferred from *Boletina* s.l.

**Table 1.** List of World species of the genus *Katatopygia* gen. n. All species are being transferred from *Boletina* Staeger. Their known distribution in faunal regions and subregions is given to the right. Abbreviations: **ORI** – Oriental Region **EN** – Eastern Nearctic subregion **WN** – Western Nearctic subregion **WP** – Western Palaearctic subregion **EP** – Eastern Palaearctic subregion.

**Species**	**Region**	**ORI**	**EN**	**WN**	**EP**	**WP**
	**# species**	**1**	**1**	**4**	**4**	**2**
*Katatopygia antica* (Garrett, 1924), comb. n.		–	–	•	–	–
*Katatopygia antoma* (Garrett, 1924), comb. n.		–	–	•	–	–
*Katatopygia erythropyga* (Holmgren, 1883), comb. n.		–	•	–	•	•
*Katatopygia hissarica* (Zaitzev & Polevoi, 2002),comb. n.		–	–	–	•	–
*Katatopygia laticauda* (Saigusa, 1968), comb. n.		•	–	–	–	–
*Katatopygia magna* (Garrett, 1925), comb. n.		–	–	•	–	–
*Katatopygia neoerythropyga* (Zaitzev & Polevoi, 2002), comb. n.		–	–	–	•	–
*Katatopygia sahlbergi* (Lundström, 1906), comb. n.		–	–	•	•	•

## Material and methods

**Figure 1.**
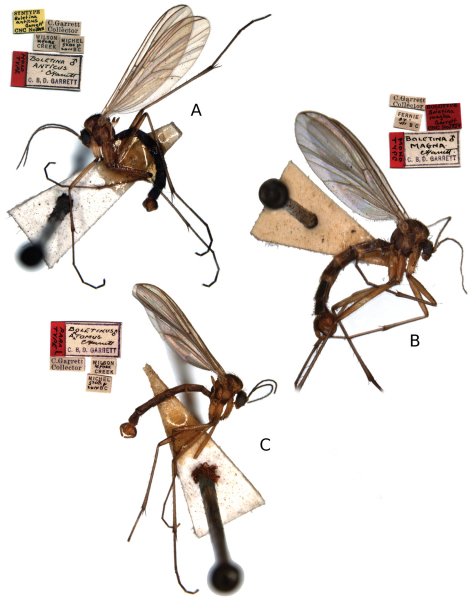
Type material with original labels of species of *Katatopygia* gen. n. described by Garrett (1924, 1925). **A** *Katatopygia antica* (Garrett, 1924) male syntype **B** *Katatopygia magna* (Garrett, 1925) male holotype. **C** *Katatopygia antoma* (Garrett, 1924) male syntype.

The examined material was gathered from museum collections and surveys, and mainly consists of the type series of the species described by CBD Garrett (Fig. 1) from Canada, some material from Alaska (USA) and European material from the Nordic region. The following collection acronyms for depositories are used in the text:

AMNH American Museum of Natural History, New York, USA

CNC Canadian National Museum, Ottawa, Canada

CUIC Cornell University, Ithaca, New York, USA

KMNH Kyushu University Museum, Fukuoka, Japan

MZLU Museum of Zoology, Lund University, Lund, Sweden

NHRS Swedish Museum of Natural History, Stockholm, Sweden

ZMUN Zoological Museum, University of Oslo, Oslo, Norway

All specimens examined were recorded with unique identification codes prefixed by “JKJ–SPM–” in a BIOTA 2.04 database (Colwell 2007), and the lists of material examined were extracted from this database. For each species and country the localities are sorted hierarchically within provinces, districts, localities and sites, respectively.

Morphological terminology mainly follows Søli (1997), the term “retinacula” is here used for any assemblage of strong, short and blunt macrosetae. The term “apical processus” is adopted from Zaitzev & Polevoi (2002) and is used for a small appendage that articulates to an unsclerotized area apically on the gonostylus. Terminology of sensillae follows Seifert (1975).

Terminalia were macerated in heated KOH (90°C) and transferred to acetic acid for neutralisation, then to alcohol and finally to glycerine. Most terminalia are preserved in glycerine in micro-vials together with the rest of the specimen, while some specimens are permanently mounted in Canada balsam on slides as outlined by Kjærandsen (2006). In order to produce plates the terminalia were either photographed in glycerine with a Nikon Digital Sight DS-M5 microscope camera mounted on a Nikon SMZ1500 stereomicroscope, or placed in alcohol gel under a coverslip and photographed through a Nikon Eclipse 50i compound microscope. Series of z-stack photos were taken and combined for extended focus using HELICON FOCUS. The images were digitally edited in ADOBE PHOTOSHOP and GIMP. Scanned sketches, drawn using a drawing tube attached to a Nikon Eclipse 50i compound microscope, were used as templates to produce digital illustration with GIMP.

### Phylogenetic analysis

A data matrix (Table 2) for phylogenetic reconstruction was constructed using WINCLADA v1.00.08 (Nixon 2002). Characters dealing with structures of the thoracic sclerites, wings, abdominal sclerites and male terminalia were used, with a focus on characters of the terminalia. The characters were either coded as binary (15) or multistate (8). Missing data was coded as “?”. All species of *Katatopygia* were included as the ingroup. Character states for *Katatopygia laticauda*, *Boletina neoerythropyga* (Zaitzev & Polevoi, 2002) and *Boletina hissarica* (Zaitzev & Polevoi, 2002) were derived from the original descriptions (Saigusa 1968; Zaitzev and Polevoi 2002). As outgroups we used *Coelosia gracilis* Johannsen, *Gnoriste longirostris* Siebke, an undescribed species of *Docosia* *cf.* *gilvipes* (Kjærandsen & Hedmark in prep.) and four species of *Boletina*, viz. *Boletina trivittata* (Meigen), *Boletina gripha* Dziedzicki, *Boletina hedstroemi* Polevoi & Hedmark and *Boletina sciarina* Staeger. The trees were rooted with the *Docosia* species. The following 23 characters were used in the analysis; observed character states are given in Table 2.

**Table 2.** Observed states of morphological characters used in the phylogenetic studies of *Katatopygia* gen. n., *Katatopygia hissarica*, *Katatopygia laticauda* and *Katatopygia neoerythropyga* are coded based on original descriptions.

	**Characters**																						
**Taxon**	**1**	**2**	**3**	**4**	**5**	**6**	**7**	**8**	**9**	**10**	**11**	**12**	**13**	**14**	**15**	**16**	**17**	**18**	**19**	**20**	**21**	**22**	**23**
*Docosia* sp. A	2	0	1	2	1	0	0	1	0	0	2	0	0	0	2	0	0	0	0	0	2	0	1
*Coelosia gracilis*	0	0	1	0	1	0	0	0	0	0	1	0	0	0	0	0	0	0	0	2	0	0	1
*Gnoriste longirostris*	1	1	1	0	0	0	1	1	0	0	2	0	2	0	0	0	0	1	?	?	0	0	1
*Boletina gripha*	0	0	1	0	0	0	1	0	0	1	1	0	0	0	0	0	0	0	0	0	2	0	1
*Boletina hedstroemi*	0	1	1	0	1	0	1	0	0	1	0	0	0	0	2	0	0	0	?	?	2	0	0
*Boletina sciarina*	0	0	1	0	0	0	1	0	0	1	0	0	0	0	2	0	0	0	?	?	2	0	0
*Boletina trivittata*	1	1	0	0	0	0	0	0	0	1	2	0	0	0	2	0	0	0	0	2	1	0	0
*Katatopygia antica*	0	1	0	0	0	1	0	1	1	0	1	2	1	1	0	1	1	1	1	1	0	1	0
*Katatopygia erythropyga*	0	1	1	1	0	1	0	1	1	0	1	0	1	1	1	1	0	1	1	1	0	0	0
*Katatopygia antoma*	0	1	0	0	0	1	0	1	1	0	1	2	1	1	0	1	1	0	1	1	0	1	0
*Katatopygia sahlbergi*	0	0,1	0	0	0	1	0	1	1	0	1	1	1	1	0	1	1	1	1	1	0	1	0
*Katatopygia laticauda*	?	0	0	0	0	1	?	?	1	0	1	1	1	1	0	1	1	1	?	?	0	0	0
*Katatopygia hissarica*	?	1	1	1	?	1	?	?	1	0	?	0	1	1	1	1	?	1	?	?	0	0	0
*Katatopygia neoerythropyga*	?	1	1	0	?	0	?	?	1	0	1	2	1	0	1	1	?	?	?	?	0	?	0
*Katatopygia magna*	0	0	1	1	0	1	0	1	1	0	1	0	1	1	1	1	0	1	1	1	0	0	0

1. Thorax with: dorsocentrals present = 0; dorsocentrals absent = 1; dispersed setae = 2.

2. Mesonotal stripes: indistinct or absent = 0; distinct = 1.

3. Costa: ending at R_5_ termination = 0; produced beyond R_5_ = 1.

4. Sc: non setose = 0; with a few apical setae = 1; mostly setose = 2.

5. CuA-stem: without setae = 0; with setae = 1.

6. Pale abdominal markings: absent = 0; present = 1.

7. Medial fold line of abdominal sternites: absent = 0; present = 1.

8. Tergite VIII: not bearing setae = 0; bearing setae = 1.

9. Male terminalia: not dorsoventrally flattened = 0; dorsoventrally flattened = 1

10. Tergite IX: small, not covering most of gonocoxites and gonostylus = 0; large, covering most of gonocoxites and gonostylus = 1.

11. Gonocoxites: separated = 0; ventrally connected, but not fused = 1; ventrally fused = 2.

12. Gonocoxites: not projected mesocaudally = 0; moderately projected mesocaudally = 1; strongly projected mesocaudally = 2.

13. Hypandrial lobe: vestigial or absent = 0; weakly sclerotized = 1; heavily sclerotized = 2.

14. Gonostylus: without apical processus = 0; with apical processus = 1.

15. Gonostylus: without strong setae on interior surface = 0; with one strong seta on interior surface = 1; with two or more strong setae on interior surface = 2.

16. Apex of gonostylus: without retinacula = 0; with retinacula = 1.

17. Parameres: without microtrichia = 0; with microtrichia = 1.

18. Parameres: paired dorsally = 0; fused into one rod dorsally = 1.

19. Sperm sac: weakly developed or hyaline = 0; well developed and scerotized = 1.

20. Gonocoxal apodeme: vestigial or absent = 0; weakly sclerotized = 1; heavily sclerotized = 2.

21. Cerci: not bearing retinacula = 0; with retinacula evenly distributed = 1; retinacula arranged in lines = 2.

22. Tergite IX: without mesial suture = 0; with mesial suture = 1.

23. Gonostylus: simple = 0; branched = 1.

The data matrix was analysed using parsimony in NONA v2.0 (Goloboff 1999) used together with WINCLADA. The analysis was carried out with a heuristic search with 10 000 replicates (mult*10 000) and 10 starting trees per replicate (hold/10), holding 1000 trees in memory (hold 1000), using the multiple TBR + TBR search strategy (mult*max*) and random starting seed (rs 0). Characters were treated as unordered and with equal weights. Jackknifing (Farris et al. 1996) were preformed in NONA v2.0 (Goloboff 1999) used together with WINCLADA in order to explore support for nodes. The analysis was carried out with 1000 jackknife replicates and 100 heuristic searches in each replicate, saving one tree per replicate.

**Figure 2.**
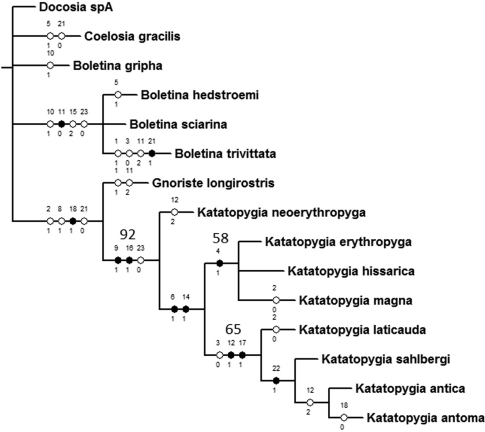
Phylogeny of *Katatopygia* gen. n. Strict consensus tree (L 53; CI 58; RI 74) calculated from the four most parsimonious trees (L 49; CI 63; RI 79) obtained with the program NONA, with all unambiguous character changes shown and unsupported nodes collapsed. Numbers above branches indicate Jackknife support above 50%. Numbers above hatch marks (black = unique, open = homoplasious) refer to characters, numbers under hatch marks refer to a state changes to the state indicated.

The heuristic search produced four most parsimonious trees (L 49; CI 63; RI 79). A strict consensus tree was calculated and is shown in Fig. 2 with all unambiguous character changes and unsupported nodes collapsed. The new genus *Katatopygia* forms a monophyletic group that is statistically supported (92%) by Jackknifing. This is in accordance with previous molecular studies (Martinsson et al. 2011) although we here end up with a different arrangement among outgroup genera and clades that gain little or no statistical support by the Jackknife analysis.

The monophyly of *Katatopygia* is supported by two unique and one non-unique synapomorphies (character and states given in parenthesis), viz.: 1) Male terminalia dorsoventrally flattened (#9:1), 2) gonostylus with apex covered with retinacula (#16:1), and 3) gonostylus simple (#23:0).

The genus *Katatopygia* has the parameres fused into one dorsal rod (#18:1) in all species except *Katatopygia antoma* where paired parameres are retained. This is interpreted as a secondary reversal and this character is here a synapomorphy shared with *Gnoriste*.

Among the *Katatopygia* species *Katatopygia neoerythropyga* is found as the sister-group to the other species. This clade is supported by two synapomorphies viz.: 1) pale abdominal markings present (#6:1) and 2) gonostylus with an apical processus (#14:1). This clade is further subdivided into two distinct clades. One includes the ‘*erythropyga*-like’ species (*Katatopygia erythropyga*, *Katatopygia hissarica* and *Katatopygia magna*)that is moderately(58 %) supported by Jackknifing and has one synapomorphy; Sc with a few apical setae (#4:1). The other clade includes the ‘s*alhbergi*-like’ species (*Katatopygia sahlbergi*, *Katatopygia antica*, *Katatopygia antoma*, and *Katatopygia laticauda*)that is moderately(65%) supported by Jackknifing and has three synapomorphies, viz.: 1) costa ending at R_5_ termination (#3:0, a character state also found in *Boletina trivittata*), 2) gonocoxites moderately projected mesocaudally (#12:1), and 3) parameres covered with microtrichia (#17:1).

The *erythropyga-*clade is unresolved, whereas the *sahlbergi*-clade is fully resolved with *Katatopygia laticauda* being the sister-group to the remaining species that are united by having tergite IX with a mesal suture (#22:1). *Katatopygia antica* and *Katatopygia antoma* share one synapomorphy; gonocoxites strongly projected mesocaudally (#12:2, this state is also found in *Katatopygia neoerythropyga*).

The data matrix and trees are deposited in the Dryad Data Repository at http://dx.doi.org/10.5061/dryad.682t7442

## Systematics

### Genus Katatopygia gen. n.

urn:lsid:zoobank.org:act:1A68AEFE-FE7E-4D92-BC9B-430B52D6979A

http://species-id.net/wiki/Katatopygia

http://sciaroidea.info/taxonomy/41708

#### Type species.

*Boletina sahlbergi* Lundström, 1906: 14(type deposited in MZHF)

#### Diagnosis.

**Figure 3.**
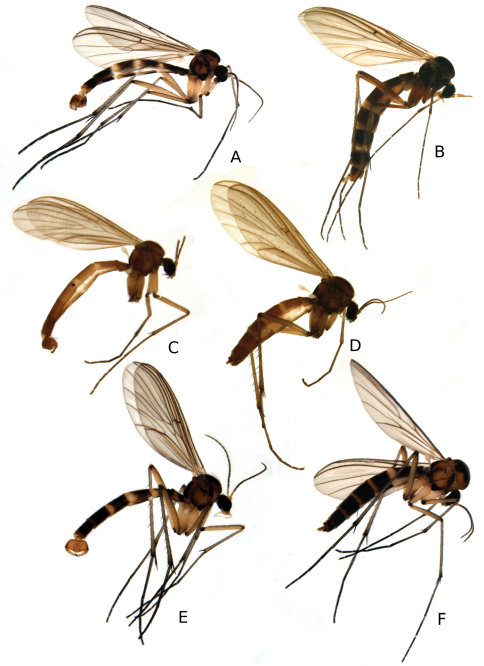
Habitus photos of species of *Katatopygia* gen. n. Lateral view **A** *Katatopygia erythropyga* (Holmgren, 1883), male **B** *Katatopygia erythropyga* (Holmgren, 1883), female. **C**. *Katatopygia antoma* (Garrettt, 1924), male **D** *Katatopygia antoma* (Garrettt, 1924), female **E** *Katatopygia sahlbergi* (Lundström, 1906), male **F** *Katatopygia sahlbergi* (Lundström, 1906), female

 The genus consists of medium sized slender Gnoristinae with long abdomen where the males have a very characteristically flattened and dilated terminalia (eg. Fig. 3E). They can be recognized on a combination of the following characters: mouthparts not prolonged; scutum with setae arranged in acrostichals, dorsocentrals and laterals; laterotergite bare; wing with Sc ending in C; M-petiole as long as or longer than ta; CuA-furcation before level of M-fork, approximately level with base of Rs; abdominal sternites with median fold-line absent; male terminalia broad and dorsoventrally flattened, often rotated about 180°; gonostylus large and simple, bearing an apical processus (except in *Katatopygia neoerythropyga*); parameres fused dorsally into one caudally directed rod (with one exception, *Katatopygia antoma*, that has pared parameres); cerci large and without retinacula, covered with long trichia; hypoproct well developed; female terminalia with unsegmented cerci. The best characters to separate between *Katatopygia* and *Boletina* s.s. are further listed in Table 3.

**Table 3.** Comparison of *Katatopygia* gen. n. and *Boletina* Staeger s. str.

Character	*Katatopygia*	*Boletina* s. str.
Laterotergite	bare	bare or setose
Median fold line on abdominal sternites	absent	present or absent
Male terminalia	broad and dorsoventrally flattened	shaped differently
Male parameres	fused (one exception *Katatopygia antoma*)	paired
Male cercus	large, not bearing retinacula	smaller, bearing retinacula
Male tergite IX	small	large
Male gonostylus	with apical processus (one exception *Katatopygia neoerythropyga*)	without apical processus
Apex of male gonostylus	bearing retinacula	not bearing retinacula
Female cercus	one segmented	two segmented (except in *Boletina abdita* and *Boletina oviducta*)

#### Description.

 Adults: Medium sized, slender with long abdomen, body length 4.5–6.5 mm (Fig. 3).

**Figure 4.**
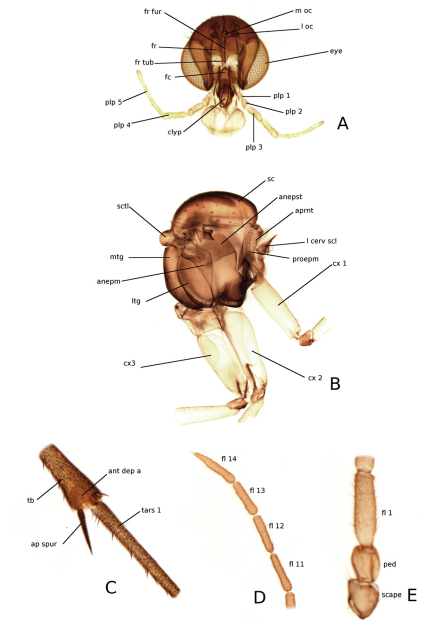
Morphology of *Katatopygia* gen. n. [*Katatopygia sahlbergi* (Lundström, 1906)] **A** Head, frontal view. **B** Thorax, lateral view **C** Front leg **D** Apex of antenna **E** Base of antenna. Abbreviations: anepist = anepisternum; anepm = anepimeron; aprnt= anteprononun; ap spur = apical spur; clyp = clypeus; cx 1 = forecoxa; cx 2 = midcoxa; cx 3 = hindcoxa; eye = compound eye; fc = face; fl =flagellar segment; fr fur = frontal furrow; fr tub = frontal tubercle; l oc = lateral ocelus; l cerv scl= lateral cervical sclerite ; ltg = laterotergite; m oc = medial ocellus; mtg = mediotergite; ped = pedicel; plp =palpomere; proepm = proepimeron; sc = scutum; sctl = scutellum; tars 1 = tarsomere one; tb = tibia.

**Head** (Fig. 4A). Vertex with scattered setae. Ocelli three, almost in line, the median slightly smaller than laterals, lateral ocelli separated from eye by approximately 1.5 times its diameter; below the ocelli, protuberances present and well sclerotized. Eyes with shallow emargination above antennal base. Frons without setae but with small microtrichia and on lateral parts some stronger microtrichia; frontal furrow well developed and reaching apex of frontal tubercle. Antenna with 14 flagellomers; scape and pedicel with a few scattered setae and short microtrichia (Fig. 4E); flagellomeres long rectangular, densely covered with medium sized setae; apical flagellomere with a somewhat stronger terminal seta (Fig. 4D). Face with scattered setae. Mouthparts not prolonged; clypeus oval to subtriangular and well separated from face, sclerotized and bearing setae; palps with five palpomeres, the first being reduced and easily overlooked, third palpomere with sensillae on inner surface.

**Thorax** (Fig. 4B). Antepronotum fused with proepisternum, bearing some setae, the suture between the sclerites weak. Scutum with setae arranged in acrostichals, dorsocentrals and laterals, otherwise bare. Scutellum with one pair of bristles and scattered setae. Anepisternum, anepimeron latero- and mediotergite all bare.

**Figure 5.**
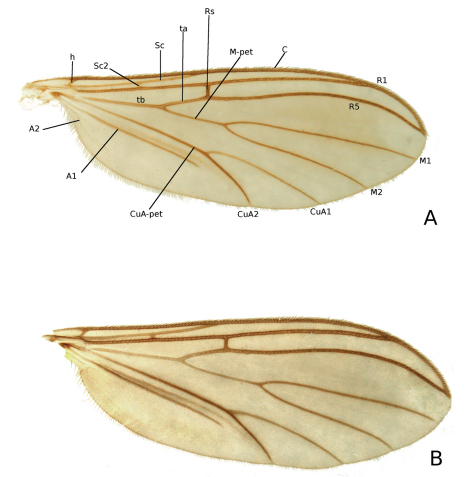
Wing photos of *Katatopygia* gen. n. **A** *Katatopygia sahlbergi* (Lundström, 1906) **B** *Katatopygia antoma* (Garrettt, 1924)] Abbreviations: A1 = anterior anal vein; A2 = posterior anal vein; C = Costal vein; CuA1 and CuA 2 = anterior branch of cubitus; CuA–pet = petiole of CuA; h = humeral; M–pet = petiole of media; M1 and M2 = branches of media; R1 = anterior branch of radius; R5 = posterior branch of radius; sc = subcosta; ta = anterior transversal (= crossvein rm); tb = basal transversal.

**Wings** (Fig. 5A–B). Wing membrane unspotted, yellow tinged with dense, irregular arranged microtrichia and no macrotrichia. Crossvein h bare; costa, R_1_, and R_5_ with both dorsal and ventral setae; M_1_, M_2_, CuA_1_ and CuA_2_ with dorsal setae; subcosta bare or with a few setae on distal part; ta, tb, M-petiole, CuA-petiole, A_1_ and A_2_ without setae; C ending in, or slightly produced beyond apex of R_5_; Sc ending in C before or in level with base of Rs; Sc_2_ present, but may be reduced; R_4_ absent; M-petiole between 1 and 2 times as long as ta; CuA-fork starts proximally of M-fork, approximately at the level of base of Rs; A_1_ ending at or slightly before CuA-fork; A_2_ indistinct and short.

**Legs** (Fig. 4C). Legs often pale with dark setation; fore and mid coxae with some setae on apical part; trochanter dark; bearing sensillae and a few small setae; femur with numerous setae and no bristles; tibia covered with irregularly arranged setae and with bristles mainly confined to ventral surface; fore tibia with anteroapical depressed area semicircular and densely covered with long microtrichia; apical tibial spur serrated and covered with microtrichia, no apical comb present; tarsus covered with macrotrichia and some stronger setae; claws with a small ventral lobe; empodium pulvilliform.

**Abdomen**. Pale abdominal markings, when present, situated towards the apices of the tergites. Sternite 1 with a few weak setae apically, all other segments haired; sternites with sublateral fold-lines, median fold-line absent; segment 7 and 8 reduced and retracted into segment 6.

**Figure 6.**
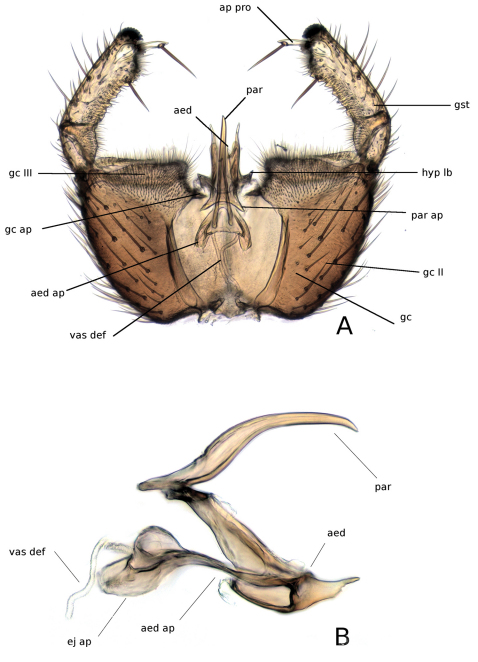
Morphology of male terminalia of *Katatopygia* gen. n. **A** Male terminaliaof *Katatopygia erythropyga* (Holmgren, 1883), dorsal view, T9 removed **B** Aedagus and parameres of *Katatopygia erythropyga* (Holmgren, 1883), lateral view. Abbreviations: **aed** = aedeagus; **aed ap**= aedeagal apodeme; **ap pro** = apical processus of gonostylus; **ej ap** =ejaculatory apodeme; **gc II** = section II of gonocoxite; **gc III** = section III of gonocoxite; **gc ap** = gonocoxal apodeme; **gst** = gonostylus; **hyp lb** = hypandrial lobe; **par** = paramere; **par ap** = parameral apodeme; **vas def** = vas deferens.

**Male terminalia** (Fig. 6A–B). Broad and dorsoventrally flattened; often rotated about 180°. Tergite IX rather small and subrectangular, in some species with a mesial sclerotized suture, scattered with setae.Cerci large, rounded to oval, without retinacula, densely covered with long microtrichia. Gonocoxites large, moderately incised ventrally with a hypandrial lobe situated in this incision; hypandrial lobe well developed and more or less branched; gonocoxite bearing scattered macro- and micotrichia, long microtrichia densely covering apical margin. Tergite X present as a weakly sclerotized, short and broad plate situated ventrally, near apex of tergite IX. Hypoproct well developed, situated ventrally to cerci and fused with tergite X, setose and resembling a second segment of cercus. Gonostylus large, unbranched except posessing a tiny apical processus which articulates to a small unsclerotized area and bears 1–2 strong setae, in some species this processus is minute or absent; apex of gonostylus covered with dense retinacula; ventrobasally surface of gonostylus with patch of placoid sensillae; inner surface of gonostylus usually fringed with small dentations. Accessory copulatory appendages joined to gonocoxite through a weakly sclerotized gonocoxal apodeme attached near apex of aedeagus. Aedeagus apically connected with parameres; in most species the parameres are fused dorsally into one caudally directed rod; aedeagus with well developed sperm sacs, to which vas deferens is attached.

**Figure 7.**
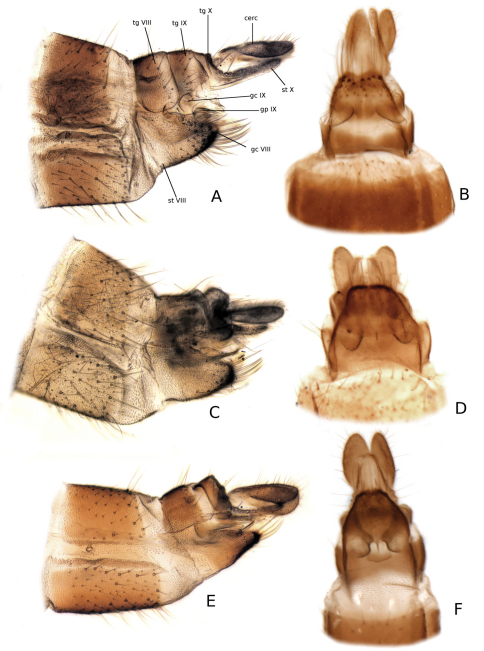
Female terminalia of *Katatopygia* gen. n. **A**, **B** *Katatopygia erythropyga* (Holmgren, 1883) **C**, **D** *Katatopygia antoma* (Garrett, 1924) **E,** **F** *Katatopygia sahlbergi* (Lundström, 1906) [A, C and E in lateral view, B, D and F in ventral view]. Abbreviations: cerc = cereus; gc =gonocoxite; gp =gonapophysis; st = sternite; tg = tergite.

**Female terminalia** (Fig. 7A–F). Tergite VIII well developed, subrectangular. Sternite VIII well developed, entirely fused with gonocoxite VIII that is tapered and bearing several strong setae at apical margin. Tergite IX well developed, shorter than Tergite VIII. Gonapophysis VIII hyaline, indistinct. Gonapophysis IX ventrally divided and retracted into segment VIII, in some species projected into a pointed apex, while in other short and blunt. Tergite X very short, laterally fused with sternite X that is completely divided ventrally and projected caudally. Cerci one-segmented, ovate.

**Larvae** unknown.

#### Notes on biology.

 The Nordic species are most abundant in boreal Taiga and subarctic environments, and are possibly strictly boreal-montane. The adults, at least of *Katatopygia sahlbergi*, seem to be attracted by light, which could suggest nocturnal activity. Larval habitats are unknown for all species in the genus.

#### Distribution.

 A mainly Holarctic genus with the exception of *Katatopygia laticauda* (Saigusa, 1968), comb. n. described from Taiwan in the Oriental Region (Saigusa 1968). The greatest diversity is found in Western North America and in the Eastern Palaearctic with four species in each of the regions (Table 1).

#### Etymology.

*Katatopygia* is derived from the Greek words *katatonis*, meaning “broader than high”, *pygo*-, meaning “rump” or “buttock” and the suffix *-ia* denoting pertaining to. The name refers to the characteristic broad and dorsoventrally flattened terminalia shared by all males in the genus. The name is a noun and is feminine.

##### The species of Katatopygia

### Katatopygia antica

(Garrett, 1924) comb. n.

http://sciaroidea.info/taxonomy/41721

Fig. 1A 8A–C

Boletina anticus Garrett, 1924: 165

#### Diagnostic characters.

Most similar to *Katatopygia antoma*, with which it shares the projected dorsomesal corners of the male gonocoxites. Distinguished from *Katatopygia antoma* by having parameres fused, a small median tooth on hypandrial lobe and brown tip of halter.

#### Re-description.

 Male. **Wing length** 5.0–5.5 mm.

**Head** brown; palps and mouthparts pale. Antenna with scape brown, pedicel and basal part of first flagellomere pale, rest of flagellum brown.

**Thorax** brown with distinct, dark brown mesonotal stripes, humeral area yellow. Antepronotum brown; anepisternum brown; preepisternum brown; laterotergite brown; mediotergite brown. Halter pale with apical part of knob brown.

**Wings** weakly brownish tinged; veins yellowish brown; stem of M approximately 1.7 times the length of ta; Sc_2_ present; Sc bare and ending in C at or slightly before base of Rs; C ending at apex of R_5_.

**Legs** pale with joints darker.

**Abdomen** dark brown often with narrow pale apical bands on tergites II-IV.

**Terminalia** brown. Gonocoxite with dorsomesal corner forming a mesocaudally directed horn-like processus, distinctly more projected than the ventromesal corner. Hypandrial lobe well developed and only shallowly emarginated medially with a small sharp medial tooth. One slender paramer, bearing microtrichia. Tergite IX subrectangular, with a sclerotized mesal suture. The apical processus on gonostylus approximately half as long as the diameter of gonostylus and slightly branched with two strong setae. Interior surface of gonostylus without strong setae.

Female. **Coloration** as in male, with brown tip of halter. **Terminalia** not studied.

**Figure 8.**
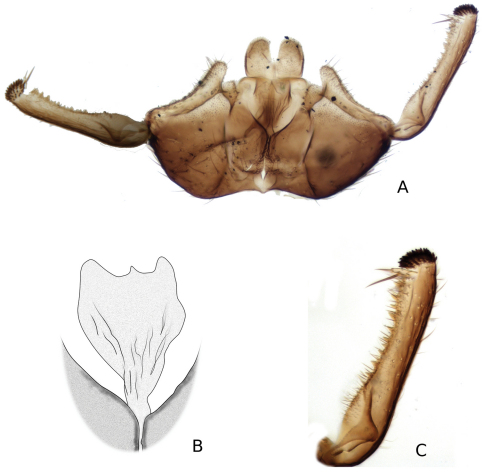
*Katatopygia antica* (Garrett, 1924) **A** Male terminalia, ventral view **B** Hypandrial lobe **C** Gonostylus, dorsal view.

#### Distribution.

 Nearctic: Canada, British Columbia.

#### Remarks. 

Only known with the type material.

#### Type material studied.

**Syntype series**. **Canada: B. C.** Michel, Wilson Creek. 21 Sep (year unknown pre 1925), leg. C. Garrett – 2 males (CNC, 1 pinned, JKJ-SPM-057739, and 1 pinned with abdomen mounted on separate slide, JKJ-SPM-057740); 24 Sep (year unknown pre 1925), leg. C. Garrett – 3 females (CNC, pinned, JKJ-SPM-057743-45).

### Katatopygia antoma

(Garrett, 1924) comb. n.

http://sciaroidea.info/taxonomy/41722

Figs 1C 3C–D 5B 7C–D 9A–G

Boletina antomus Garrett, 1924:166

#### Diagnostic characters.

Most similar to *Katatopygia antica*, with which it shares the projected dorsomesal corners of the male gonocoxites. Distinguished from *Katatopygia antica* by having two parameres, hypandrial lobe without median tooth and pale halter.

#### Re-description.

 Male. Wing length 4.5 mm.

**Head** brown; palps and mouthparts pale. Antenna with scape, pedicel and basal part of first flagellomere pale, rest of flagellum brown.

**Thorax** pale with 3 distinct, dark brown mesonotal stripes on yellow ground, humeral area pale. Mediotergite with a darker central stripe; preepisternum darker ventrally. Halter whitish.

**Wings** weakly brownish tinged; stem of M approximately 1.9 times the length of ta; Sc_2_ present; Sc bare and ending in C slightly before base of Rs. C ending in apex of R_5_.

**Legs** pale brown.

**Abdomen** brown often with narrow pale bands on tergites II-III.

**Terminalia** brown. Gonocoxite with dorsomesal corner forming a mesocaudally directed horn-like processus, distinctly more projected than the ventromesal corner. Hypandrial lobe well developed and only shallowly emarginated medially, without medial tooth. Two slender parameres, bearing microtrichia. Tergite IX subrectangular, with a sclerotized mesal suture. The apical processus on gonostylus approximately half as long as the diameter of gonostylus and slightly branched with two strong setae. Interior surface of gonostylus without strong setae.

Female. **Coloration** as in male except pale apical bands on tergite II-V.

**Terminalia**. Tergite VIII broad with rounded apicolaterally margin; sternite VIII and gonocoxite VIII short and broad with about 6 strong apical setae; gonapophysis IX long and projected into a pointed apex.

**Figure 9.**
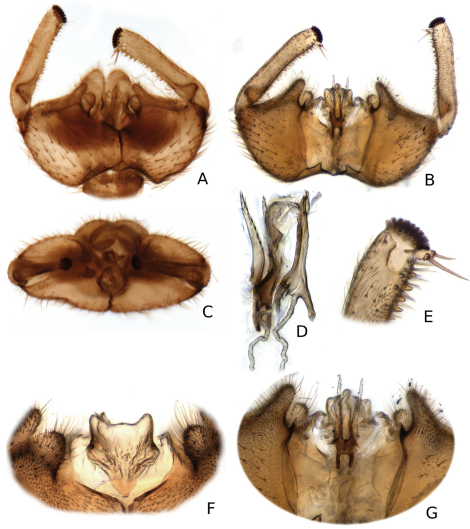
*Katatopygia antoma* (Garrettt, 1924). **A** Male terminalia, ventral view **B** Male terminalia, dorsal view with tergite 9 removed **C** Male terminalia, caudal view **D** Aedeagus and parameres, lateral view **E** Apex of gonostylus, dorsal view **F**. Hypandrial lobe **G** Aedeagus and parameres dorsal view.

#### Distribution.

 Nearctic, known from Canada, British Columbia and USA, Alaska.

#### Type material studied.

**Syntype series**. **Canada: BC,** Cranbrook, 24 Sep 1922, leg. C. Garrett – 1 female (CNC, pinned, JKJ-SPM-057751); Michel, Wilson Creek. 1 Sep (year unknown pre 1925), leg. C. Garrett – 1 female (CNC, pinned, JKJ-SPM-057753); 24 Sep (year unknown pre 1925), leg. C. Garrett – 3 males (CNC, 1 pinned with cleared terminalia in glycerine, JKJ- SPM-057747, 2 pinned, JKJ- SPM-057748-49), 4 females (CNC, pinned, JKJ-SPM-057755&59-61); 27 Sep (year unknown pre 1925), leg. C. Garrett – 1 female (CNC, pinned, JKJ-SPM-057756); 28 Sep (year unknown pre 1925), leg. C. Garrett – 1 female (CNC, pinned, JKJ-SPM-057757); 2 Oct (year unknown pre 1925), leg. C. Garrett (CNC, pinned, JKJ-SPM-057750); locality and date unknown, marked T.112 – 1 female (CNC, pinned, JKJ-SPM-057752); locality and date unknown, labelled 1815 – 1 female (CNC, pinned, JKJ- SPM-057758).

#### Other material studied.

**USA**: **Alaska**, Palmer, 13 Jul 1964 (Leg. K. M. Sommerman) – 2 males, 1 female (MZLU, in alcohol, JKJ-SPM-034388-89).

### Katatopygia erythropyga

(Holmgren, 1883) comb. n.

http://sciaroidea.info/taxonomy/41709

Figs 3A–B 6A–B 7A–B 10A–G

**Figure 10.**
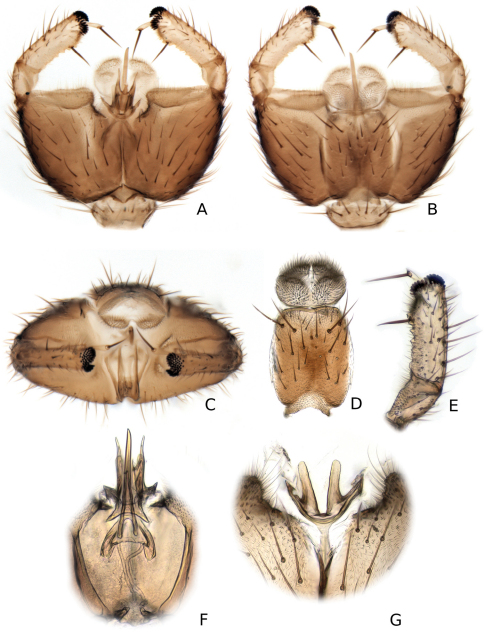
*Katatopygia erythropgya* (Holmgren, 1883) **A** Male terminalia, ventral view **B** Male terminalia, dorsal view **C** Male terminalia, caudal view **D** Tergite 9 and cerci, dorsal view **E** Gonostylys, dorsal view **F** Aedagus and paramere, dorsal view **G** Hypandral lobe.

Boletina erythropyga Holmgren, 1883:189
Boletina longicornis Johannsen, 1911:272
Boletina notescens Johannsen, misident. in Zaitzev 1994:223 fig. 74:2
Boletina erythropyga Zaitzev & Polevoi 2002:640 figs 2, 4–6, 10

#### Diagnostic characters.

 Most similar to *Katatopygia magna* and *Katatopygia hissarica*, but can be distinguished by having distinct and separated mesonotal stripes and on the evenly broad male gonostylus on which the inner dentations are reaching the basal curve.

#### Re-description.

 Male. Wing length 5.5 mm.

**Head** blackish brown; mouthparts and palps yellow. Antenna with scape, pedicel and basal part of first flagellomere pale yellow, rest of flagellum brown.

**Thorax** with three distinct, black mesonotal stripes on yellow ground, humeral area yellow; antepronotum pale; anepisternum brown; preepisternum pale with ventral half darker, brown; laterotergite brown with anterior part paler; mediotergite pale with a broad dark central stripe. Halter pale.

**Wing** pale with veins yellowish brown; M-petiole approximately 1.5 times the length of ta; Sc_2_ present; Sc ending in C slightly before Rs; Sc bearing a few setae on apical portion; C ending beyond apex of R_5_.

**Legs** pale yellow with joints darker.

**Abdomen** dark brown with yellow apical bands on tergites II–VI.

**Terminalia** brownish. Gonocoxite with mesal corners not projected; gonostylus evenly broad, angled inwards about 40° and bearing one strong seta on interior surface, dentations on interior surface reaching curve. Apical processus approximately as long as the diameter of gonostylus, bearing one apical seta. Hypandrial lobe deeply forked with four lobes. Dorsal fused paramere rod long, slender and without microtrichia. Tergite IX subrectangular, with 4 stronger setae on apical part and without a sclerotized mesial suture.

Female. Body length 6.0 mm; wing length 5.5 mm. **Coloration** as male.

**Terminalia**. Tergite IX broad with sharp apicolateral corner; gonocoxite VIII slightly incised ventrally bearing many strong apical setae; gonapophysis IX short and blunt.

#### Distribution.

 Holarctic, with records from north-western USA (Idaho) (Johannsen 1911), northern Europe (Scandinavia and northern parts of European Russia) (Zaitzev and Polevoi 2001) and Novaya Zemlya (Holmgren 1883).

#### Remarks.

 The species has been confused with *Katatopygia sahlbergi* and there are some records e.g. from the Alps and Siberia that at least partly refer to the latter species (Zaitzev and Polevoi 2001).

#### Type material.

 We were not able to locate the holotype of *Boletina longicornis* in the Johannsen collection at Cornell University (CUIC), nor can it be found in American Museum of Natural History (AMNH) (V. Blagoderov pers. com.) and should probable be regarded as lost.

#### Material examined.

**NORWAY: FV**, Alta, Elby, Valsetmoen, sandy slope, 10 Jun-6 Jul 1995 (ZMUN, leg. L.O. Hansen & H. Rinden) – 1 male. **SWEDEN: LU**, Jokkmokk, Messaure, 2 Sep-4 Oct 1971 (MZLU, leg. K. Möller) – 3 males; Luottåive NR, 28 km S Jokkmokk, 14 Jul-18 Aug 2004 (MZLU, leg. K. Hedmark & J. Kjærandsen) – 1 male; Gällivare, Haapavaara/Annavaara, 8 km WNW Vettasjärvi, 1 Jun-26 Jul 1994 (MZLU, leg. R. Rova) – 1 female, 3 male; **SÖ**, Haninge, Tyresta National Park, 19 Jun-28 Jul 2000 (NHRS , leg. B. Viklund) – 1 male; **TO**, Kiruna, Abisko, 150–500 m W Naturv. stn., 18–25 Aug 1975 (MZLU, leg. K. Möller) – 1 male; LF-05, 150–500 m W Naturv. stn., 28 Jun-5 Jul 1976 (MZLU, leg. K. Möller) – 2 males; above tree limit 26 Jun-15 Jul 2006 (NHRS, Leg. Swedish Malaise Trap Project) – 1 female, 10 males; **VB**, Skellefteå, Stenträsk, Björnhultet Domänreservat, 17 May-17 Oct 1997 (NHRS, leg. B. Viklund) – 4 males.

### Katatopygia hissarica

(Zaitzev & Polevoi, 2002) comb. n.

http://sciaroidea.info/taxonomy/41710

Boletina hissarica Zaitzev & Polevoi, 2002: 640 (figs 1, 3 & 9)

#### Diagnostic characters

.Very similar to *Katatopygia erythropyga*, from which it can be separated only on details of the male terminalia. Zaitzev & Polevoi (2002) used four key characters to distinguish them: 1) Apical process of the gonostylus slightly bolder and with more developed unsclerotized area around base of this process; 2) Dentations on the inner surface of gonostylus being restricted to distal part, not reaching the curve basally; 3) Apical part of tergite IX less sclerotized; 4) Details of aedeagus as figured by them.

#### Distribution.

 The species is known only from the holotype from Tadzhikistan.

#### Remarks.

 The species limit between *Katatopygia erythropyga* and *Katatopygia hissarica* seems vague, and it is possibly that *Katatopygia hissarica* will fall inside the variation of *Katatopygia erythropyga* when a wider range of material is studied.

#### Type material.

 The holotype is deposited in the Zoological Institute in St. Petersburg, Russia – not studied.

### Katatopygia laticauda

(Saigusa, 1968) comb. n.

http://sciaroidea.info/taxonomy/41790

Boletina laticauda Saigusa, 1968: 4 (figs 1–4)

#### Diagnostic characters.

Very similar to*Katatopygia sahlbergi* from which it can be separated by the following characters (from Saigusa 1968, table 2): 1) wing vein Sc_2_ absent; 2) wing veins M-pet and ta of approximately the same length; 3) scutum with black mesonotal stripes fused; 4) thoracic pleura entirely black; 5) middle and hind coxae black.

#### Distribution.

 Taiwan, only known with the holotype.

#### Type material.

 The holotype is deposited in the Kyushu University Museum, Japan – not studied.

### Katatopygia magna

(Garrett, 1925) comb. n.

http://sciaroidea.info/taxonomy/41796

Figs 1B 11A–D

Boletina magna Garrett, 1925: 5

#### Diagnostic characters.

*Katatopygia magna* is most similar to *Katatopygia erythropyga* and *Katatopygia hissarica*, but can be distinguished by having fused mesonotal stripes and on the apically broadened gonostylus on which the inner dentations are reaching the basal curve.

#### Re-description.

 Male. Wing length 6.0 mm.

**Head** blackish brown; palps yellow. Antenna with scape brown, pedicel and flagellum yellow.

**Thorax** with black mesonotal stripes fused on yellow ground, humeral area yellow; antepronotum brown; anepisternum brown; preepisternum dark with a diffuse pale spot; laterotergite brown; mediotergite dark. Halter pale.

**Wing** pale with veins yellowish brown; M-petiole approximately 1.8 times the length of ta; Sc_2_ present; Sc ending in C clearly before Rs; Sc bearing a few setae on apical portion; C ending beyond apex of R_5_.

**Legs** pale yellow with joints darker.

**Abdomen** dark brown with yellow apical bands on tergite II–IV.

**Terminalia** yellowish. Gonocoxite with mesal corners not projected; gonostylus with broadened apex; gonostylus angled inwards about 65° and bearing one strong seta on interior surface, dentations on interior surface reaching curve. Apical processus approximately as long as the diameter of gonostylus and bearing one subapical seta. Hypandrial lobe deeply forked with four lobes. Dorsal fused paramere rod long and straight, without microtrichia. Tergite IX subrectangular, without sclerotized mesial suture.

Female unknown.

**Figure 11.**
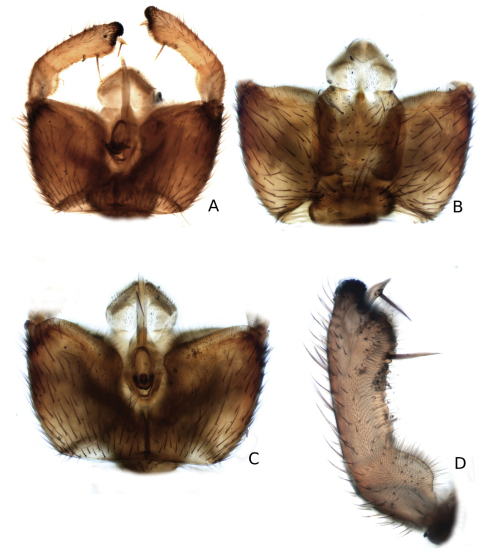
*Katatopygia magna* (Garrett, 1925) **A** Male terminalia, ventral view **B** Male terminalia, dorsal view, gonostylus omitted **C** Male terminalia, caudal view, gonostylus omitted **D** Gonostylus, dorsal view.

#### Distribution.

 Nearctic: Canada, British Columbia.

#### Remarks.

 Known only from the holotype.

#### Type material studied.

**Holotype male**. **Canada: BC**, Fernie, 24 Jul (year unknown pre 1925), leg. C. Garrett (CNC, pinned with cleared terminalia in glycerine in microtube on same pin, JKJ-SPM-057738).

### Katatopygia neoerythropyga

(Zaitzev & Polevoi, 2002) comb. n.

http://sciaroidea.info/taxonomy/41711

Boletina neoerythropyga Zaitzev & Polevoi, 2002: 641 (figs 7–8)

#### Diagnostic characters.

 Most similar to *Katatopygia antica* and *Katatopygia antoma* from which it can be separated on coloration and details of the male terminalia. Zaitzev & Polevoi (2002) used four key characters to distinguish it from *Katatopygia erythropyga*: 1) absence of the apical process of the male gonostylus; 2) longer stem of M-fork; 3) scape of antenna brown; 4) abdomen uniformly brown.

#### Distribution.

 The species is known only from the Yamal peninsula north in West Siberia.

#### Remarks.

 The absence of the apical process of the male gonostylus is unique among the known species of *Katatopygia* and may be regarded as a secondary reduction (see discussion of phylogeny).

#### Type material.

 The holotype is deposited in the A.N. Severtzov Institute of Ecology and Evolution in Moscow, Russia – not studied.

### Katatopygia sahlbergi

(Lundström, 1906) comb. n.

http://sciaroidea.info/taxonomy/41712

Figs 3E–F 4A–E 5A 7E–F 12A–F

Boletina sahlbergi Lundström 1906: 14, fig 8
Boletina punctus Garrett 1925: 5 syn. n.
Boletina altaica Zaitzev 1994: 203
Boletina sahlbergi Lundström 1912:20, figs 22–24
Boletina sahlbergi Zaitzev & Polevoi 2002:642, fig. 11

#### Diagnostic characters.

Most similar to *Katatopygia laticauda* from which it can be distinguished by having wing vein Sc_2_ present and M-pet longer than ta, and in coloration with distinct and separated mesonotal stripes and paler coxae where at most hind coxa are darkened.

#### Re-description.

 Male. Body length 4.5-6.5 mm; wing length 4.5-6.0 mm.

**Head** blackish brown; mouthparts and palps yellow. Antenna with scape, pedicel, and basal part of first, in some specimens the whole first and basal part of second, flagellomere pale yellow, rest of flagellum brown.

**Thorax** in most specimens with 3 distinct, black mesonotal stripes on yellow ground, humeral area yellow, a few specimens with mesonotal stripes indistinct and humeral area brownish; antepronotum pale; anepisternum brown; preepisternum pale with ventral half darker, brown; laterotergite brown with anterior part paler; mediotergite pale with a broad dark central stripe. Halter pale.

**Wing** pale with veins yellowish brown; stem of M approximately 1.5 times the length of ta; Sc_2_ present; Sc ending in C slightly before Rs; C ending at apex of R_5_.

**Legs** pale yellow with joints darker.

**Abdomen** dark brown, usually with yellow apical bands on tergite I–IV.

**Terminalia** often yellow with dark lateral markings, in some specimens brownish and not distinctly paler than rest of abdomen. Gonocoxite with mesal corners slightly projected. Tergite IX subrectangular, with a sclerotized mesal suture. Paramere simple, strong, blunt and covered with microtrichia. Gonostylus straight with apical processus approximately half as long as the diameter of gonostylus.

Female. Body length 6.0-6.5 mm; wing length 6.0–6.5 mm.

**Coloration** as male.

**Terminalia**. Tergite IX short with rounded apicolateral corner; sternite VIII and gonocoxite VIII long and narrow, bearing about 6 strong apical setae; gonapophysis IX long and projected into a pointed apex.

**Figure 12.**
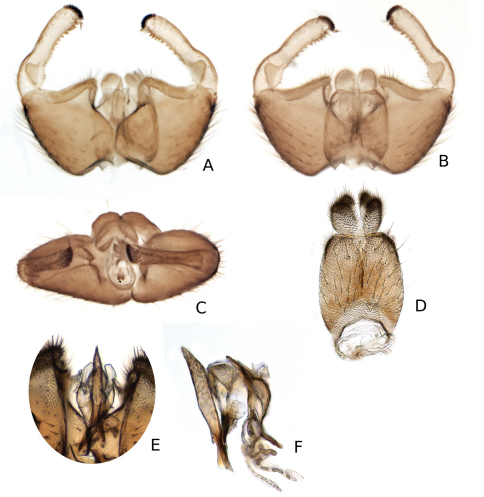
*Katatopygia sahlbergi* (Lundström, 1906) **A** Male terminalia, ventral view **B** Male terminalia, dorsal view, tergite 9 removed **C** Male terminalia, caudal view **D** Tergite 9 and cerci, dorsal view **E** Aedeagus and parameres dorsal view **F** Aedeagus and parameres, lateral view.

#### Distribution.

 Holarctic. Possibly boreal-alpine with records from Scandinavia, northern parts of European Russia, The Alps, West Siberia (Zaitzev & Polevoi 2002), Japan (Saigusa 1968) and Canada (Garrett 1925).

#### Remarks.

 The proposed synonymy of *Katatopygia sahlbergi* and *Boletina punctus* is based on the study of type material of *Boletina punctus* and Nordic material of *Katatopygia sahlbergi*. *Katatopygia sahlbergi* has been confused with *Katatopygia erythropyga* and there are some records of the latter species that at least partly refer to *Katatopygia sahlbergi* (Zaitzev & Polevoi 2002).

#### Material examined.

**NORWAY: FV**, Alta, Detsika, Buolamalia, 6 Aug-25 Sep 1996 (ZMUN, leg. L. O. Hansen & H. Rinden) – 2 males; **STI**, Oppdal, Kongsvoll, 19-26 Jul 1995 (MZLU, leg. J. Skartveit) – 1 male; Kongsvoll, Sprænbekken, 16 Aug-19 Sep 1994 (MZLU, leg. J. Skartveit) – 1 male; **SWEDEN: LU**, Jokkmokk, Kaltisbäcken 1 km NNE Messaure, 21 Jun-12 Jul 2004 (MZLU, leg. J. Kjærandsen & K. Hedmark) – 1 female, 10 males; 12 Jul-17 Aug 2004 (MZLU, leg. J. Kjærandsen & K. Hedmark) – 3 males; above parking lot, 12 Oct 1997 (MZLU, leg. S. Lundberg) – 1 male; Messaure, 2 Sep-4 Oct 1971 (MZLU, leg. K. Möller) – 14 males; Porsitjärn/Porsi VVO, 1.5 km SE Vuollerim, 6 May-13 Aug 2004 (MZLU, leg. M. Karström) – 2 females, 2 males; 15 Jun-1 Jul 2005 (MZLU, leg. K. Hedmark & M. Karström) – 2 males; 1-16 Jul 2005 (MZLU, leg. K. Hedmark & M. Karström) – 1 male; Tapmokbäckravinen, 12 km SSE Vuollerim, 16 Jun 2004 (MZLU, leg. J. Kjærandsen) – 1 male; Bombmurkleskogen VVO, 4 km SSE Messaure, 17 Jun 2004 (MZLU, leg. J. Kjærandsen) – 1 male; 21 Jun 2004 (MZLU, leg. J. Kjærandsen) – 1 male; 7-19 Jul 2005 (NHRS, leg. Swedish Malaise Trap Project) – 2 males; Luottåive NR, 28 km S Jokkmokk, 14 Jul-18 Aug 2004 (MZLU, leg. K. Hedmark & J. Kjærandsen) – 2 males; 18 Aug-20 Sep 2004 (MZLU, leg. K. Hedmark & J. Kjærandsen) – 4 females, 2 males; Gällivare, Haapavaara/Annavaara, 8 km WNW Vettasjärvi, 1 Jun-26 Jul 1994 (NHRS, leg. R. Rova) – 1 male; Jokkmokk, Bombmurkleskogen VVO, 9-25 Sep 2005 (NHRS, leg. Swedish Malaise Trap Project) – 1 male; 25 Sep-13 Oct 2005 (NHRS, leg. Swedish Malaise Trap Project) – 1 female, 3 males; **TO**, Kiruna, Abisko, 14-20 Jul 1975 (MZLU, leg. K. Möller) – 4 males; GF-02, 150-500 m W Naturv. stn., 10-25 Jul 1975 (MZLU, leg. K. Möller) – 1 male; LF-01, 150-500 m W Naturv. stn., 6-20 Jun 1975 (MZLU, leg. K. Möller) – 2 males; 22-29 Sep 1975 (MZLU, leg. K. Möller) – 1 male; 29 Sep-6 Oct 1975 (MZLU, leg. K. Möller) – 1 male; LF-02, 150-500 m W Naturv. stn., 21-28 Jul 1975 (MZLU, leg. K. Möller) – 1 male; 28 Jul-4 Aug 1975 (MZLU, leg. K. Möller) – 2 males; 29 Sep-6 Oct 1975 (MZLU, leg. K. Möller) – 1 male; LF-03, 150-500 m W Naturv. stn., 25 Sep-6 Oct 1975 (MZLU, leg. K. Möller) – 13 males; 6-20 Oct 1975 (MZLU, leg. K. Möller) – 2 males; 20-27 Oct 1975 (MZLU, leg. K. Möller) – 1 male; LF-04, 150-500 m W Naturv. stn., 22-29 Sep 1975 (MZLU, leg. K. Möller) – 1 female, 2 males; LF-05, 150-500 m W Naturv. stn., 6-20 Oct 1975 (MZLU, leg. K. Möller) – 1 female, 11 males; 20-27 Oct 1975 (MZLU, leg. K. Möller) – 1 male; LF-06, 150-500 m W Naturv. stn., 4-11 Aug 1975 (MZLU, leg. K. Möller) – 1 male; 1-8 Sep 1975 (MZLU, leg. K. Möller) – 1 male; 15-22 Sep 1975 (MZLU, leg. K. Möller) – 3 males; 22-29 Sep 1975 (MZLU, leg. K. Möller) – 2 females, 11 males; 29 Sep-6 Oct 1975 (MZLU, leg. K. Möller) – 24 males; LF-07, 150-500 m W Naturv. stn., 8-15 Sep 1975 (MZLU, leg. K. Möller) – 1 male; 29 Sep-6 Oct 1975 (MZLU, leg. K. Möller) – 14 males; 6-20 Oct 1975 (MZLU, leg. K. Möller) – 2 males; LF-08, 150-500 m W Naturv. stn., 1-8 Sep 1975 (MZLU, leg. K. Möller) – 1 male; LF-09, 150-500 m W Naturv. stn., 21-28 Jul 1975 (MZLU, leg. K. Möller) – 1 male; 8-15 Sep 1975 (MZLU, leg. K. Möller) – 1 male; 22-29 Sep 1975 (MZLU, leg. K. Möller) – 4 males; 29 Sep-6 Oct 1975 (MZLU, leg. K. Möller) – 14 males; 6-20 Oct 1975 (MZLU, leg. K. Möller) – 1 female, 10 males; LF-10, 150-500 m W Naturv. stn., 22-29 Sep 1975 (MZLU, leg. K. Möller) – 2 males; LSF, 15 Aug-1 Sep 1975 (MZLU, leg. K. Möller) – 1 male; 1-15 Sep 1975 (MZLU, leg. K. Möller) – 2 males; Abisko, Stordalen NR, 9-24 Jul 1975 (MZLU, leg. K. Möller) – 5 males; 7-14 Aug 1975 (MZLU, leg. K. Möller) – 3 males; 4-11 Sep 1975 (MZLU, leg. K. Möller) – 2 males; above tree limit 26 Jun.-15 Jul. 2006 (NHRS, leg. Swedish Malaise Trap Project) – 4 males.

#### Type material of 

***Boletina punctus***

####  examined.

**Holotype** male. **Canada: BC**, Creston 4 Jul (year unknown pre 1926), leg B. C. D. Garrett (CNC, pinned with terminalia mounted on separate slide, JKJ-SPM-057764). **Paratypes**. Same data as holotype, marked as **allotype** – 1 female (CNC,pinned, JKJ- SPM-057778), same data as holotype – 3 females (CNC, pinned, 1 with abdomen in glycerine in microtube on same pin, JKJ- SPM-057779-81), 13 males (CNC, pinned, 2 with terminalia mounted on separate slide, JKJ-SPM-057765-77).

##### Key to the males of Katatopygia

The key is partly based on the key from Zaitzev and Polevoi (2002).

**Table:** 

1	Gonostylus with distinct apical processus (cf. Fig. 6A). Abdominal tergites often with pale apical bands	2
–	Gonostylus without distinct apical processus (Zaitzev and Polevoi 2002: fig. 7). Abdomen uniformly brown	*Katatopygia neoerythropyga*
2	Costa extending clearly beyond R_5_-termination. Gonostylus curved inwards, with strong seta on interior surface (cf. Fig. 10E). Sc with a few setae on apical portion. Parameres fused and bare (Fig. 6B)	3
–	Costa ending at or slightly beyond R_5_-termination (Fig. 5A–B). Gonostylus straighter, without strong seta on interior surface (cf. Fig. 8C). Sc bare. Parameres forked or fused, covered with microtrichia (cf. Figs 9G, 12F)	5
3	Dentations on the inner surface of gonostylus reaching the curve. Apical part of tergite IX well sclerotized	4
–	Dentations on the inner surface of gonostylus restricted to distal part, not reaching the curve (Zaitzev and Polevoi 2002: fig. 1). Apical part of tergite IX weakly sclerotized (Zaitzev and Polevoi 2002: fig. 9)	*Katatopygia hissarica*
4	Gonostylus broadened apically (Fig. 11D). Scutum with dark mesonotal stripes fused	*Katatopygia magna*
–	Gonostylus evenly broad (Fig. 10E). Scutum with dark mesonotal stripes distinct and separated	*Katatopygia erythropyga*
5	Mesodorsal corners of gonocoxite distinctly projected caudally (cf. Fig. 9A). Hypandrial lobe only shallowly emarginated (Figs 8B, 9F). Parameres forked or fused	6
–	Mesodorsal corners of gonocoxite not projected (Fig. 12B). Hypandrial lobe deeply divided mesially. Parameres fused into single rod	7
6	Dorsal part of parameres split into two processes caudally (Figs 9D, G). Hypandrial lobe without a small sharp medial tooth (Fig. 9F). Halter pale (Figs 1C, 3B)	*Katatopygia antoma*
–	Dorsal part of parameres fused into one rod caudally. Hypandrial lobe with a small sharp medial tooth (Fig. 8B). Tip of halter brown (Fig. 1A)	*Katatopygia antica*
7	M-pet and ta of approximately the same length. Sc_2_ absent (Saigusa 1968: plate 1:1). Scutum with black mesonotal stripes fused. Middle and hind coxae black	*Katatopygia laticauda*
–	M-pet longer than ta. Sc_2_ usually present (Fig. 5A). Scutum pale with dark mesonotal stripes distinct and separated, scutum rarely more uniformly brown. At most hind coxa darkened	*Katatopygia sahlbergi*

## Discussion

Resolving phylogeny of the extended Gnoristinae clade is way beyond the scope of this study and the quantitative phylogenies that have been presented so far (e.g. Söli 1997; Baxter 1999; Rindal et al. 2009; Martinsson et al. 2011) are partly conflicting and not very convincing when it comes to stable intergeneric relationships. With some 160 species placed in the rather heterogeneous genus *Boletina* we still think that it will benefit from being split into subsets of putatively natural entities, and that these taxa will shed new light on the phylogeny of the entire group. The process was initiated by Martinsson et al. (2011) who estimated the first molecular phylogenies focused on *Boletina* and related genera. The decision to erect the new genus *Katatopygia* rest largely on the supportive results from Martinsson et al. (2011) where the two European species consistently and with support across different genes were found as the basal sister-group to all the other species of *Boletina*, *Coelosia* and *Gnoriste* included in the analysis. The unequivocal basal position makes it rather unlikely that our splitting will render *Boletina* s.s. paraphyletic with respect to *Katatopygia*. The idea to segregate species of the *Boletina erythropyga/punctus*-group from *Boletina* s.l. is, however, not new and grew out of a long-funded distinct impression that these species form a morphological uniform group with highly specialized male terminalia that don’t naturally fit together with the remaining *Boletina* s.s.

The additional morphological analysis presented here was designed to test the monophyly of the extended group of eight *Katatopygia* species and resolve their interrelationships. The characters included in the analysis were thus chosen mainly for resolving relationships among *Katatopygia* species, not to resolve relationships among Gnoristinae genera. A few outgroup taxa were, based on the available phylogenies (Søli 1997; Rindal et al 2009; Martinsson et al. 2011), selected among genera available to us that have been indicated to be closely related to *Boletina* s.l. (including *Katatopygia*). Given the rather limited selection of informative characters found and the few outgroup taxa included the present analysis cannot be given the same credit to support the segregation of *Katatopygia* as did the molecular study (Martinsson et al. 2011). Focused mainly on terminalia morphology the analysis mainly summarizes those characters we found to be diagnostic for the new genus and gives a first clue to inter-relationships among its species. Accordingly the morphological analysis retrieved a monophyletic *Katatopygia* with high support whereas relationships among the outgroup taxa are not supported and somewhat contrasting those found by Martinsson et al. (2011).

The segregation of *Katatopygia* rise new questions related to the increasing number of *Boletina* “look-alike” genera. Are they forming a monophyletic clade together with *Boletina* s.s. or rather constitute an assemblage of less related plesiomorphous genera? Another recently described genus, *Heamesphaerenotus* Saigusa from China (Saigusia 2007), show some affinities with *Katatopygia* in general appearance and they have some possibly apomorphic characters in common (retinacula on male gonostylus and one-segmented female cercus). But like for *Katatopygia*, *Heamesphaerenotus* also show a number of unique apomorphies, notably the expanded eave-like mesonotum (Saigusa 2007) not seen in *Katatopygia*. Unlike species of *Boletina* s.s. species of *Aglaomiya*, *Heamesphaerenotus*, *Katatopygia* and *Saigusaia* all have some form of retinacula on the apical part of the gonostylus, and this can also be found among species of *Synapha*. The females of *Saigusaia* and *Synapha* have two-segmented cerci (e.g. Saigusa 1968) whereas all known females of *Katatopygia* and *Heamesphaerenotus* have one-segmented cerci. In fact, *Saigusaia* seems to be much closer *Synapha* than to *Boletina* (Søli 1997; Martinsson et al. 2011) and the general outline of the male terminalia among species of *Saigusaia* (see e.g. Niu et al. 2008) very much resembles that of some *Synapha* (see e.g. Kallweit and Martens 1995).

Even after the segregation of *Katatopygia*, *Boletina* s.s. remain as a large and somewhat heterogeneous genus. It is noteworthy that the type-species of the genus, *Boletina trivittata*, may also form a separate clade (Martinsson et al. 2011) including some 10 morphological similar species. Some aberrant species currently included in *Boletina*, e.g. *Boletina abdita* Plassmann, *Boletina anderschi* (Stannius) and *Boletina ovata* (Garrett), needs further studies to see if they fit within a restricted definition of the genus.

## Supplementary Material

XML Treatment for Katatopygia

XML Treatment for Katatopygia antica

XML Treatment for Katatopygia antoma

XML Treatment for Katatopygia erythropyga

XML Treatment for Katatopygia hissarica

XML Treatment for Katatopygia laticauda

XML Treatment for Katatopygia magna

XML Treatment for Katatopygia neoerythropyga

XML Treatment for Katatopygia sahlbergi
